# Crosslink between SARS-CoV-2 replication and cystic fibrosis hallmarks

**DOI:** 10.3389/fmicb.2023.1162470

**Published:** 2023-05-11

**Authors:** Virginia Lotti, Anna Lagni, Erica Diani, Claudio Sorio, Davide Gibellini

**Affiliations:** ^1^Microbiology Section, Department of Diagnostic and Public Health, University of Verona, Verona, Italy; ^2^General Pathology Section, Department of Medicine, University of Verona, Verona, Italy

**Keywords:** SARS-CoV-2, COVID-19, cystic fibrosis, CFTR, viral infections

## Abstract

SARS-CoV-2, the etiological cause of the COVID-19 pandemic, can cause severe illness in certain at-risk populations, including people with cystic fibrosis (pwCF). Nevertheless, several studies indicated that pwCF do not have higher risks of SARS-CoV-2 infection nor do they demonstrate worse clinical outcomes than those of the general population. Recent *in vitro* studies indicate cellular and molecular processes to be significant drivers in pwCF lower infection rates and milder symptoms than expected in cases of SARS-CoV-2 infection. These range from cytokine releases to biochemical alterations leading to morphological rearrangements inside the cells associated with CFTR impairment. Based on available data, the reported low incidence of SARS-CoV-2 infection among pwCF is likely a result of several variables linked to CFTR dysfunction, such as thick mucus, IL-6 reduction, altered ACE2 and TMPRSS2 processing and/or functioning, defective anions exchange, and autophagosome formation. An extensive analysis of the relation between SARS-CoV-2 infection and pwCF is essential to elucidate the mechanisms involved in this lower-than-expected infection impact and to possibly suggest potential new antiviral strategies.

## 1. Introduction

### 1.1. Cystic fibrosis: from disease to molecular alteration

Cystic fibrosis (CF) is the most common autosomal recessive disease affecting Caucasian. CF is caused by mutations in a single gene, the Cystic Fibrosis Transmembrane Conductance Regulator (CFTR) gene (Collins, 1992), which was first described in 1989 (Riordan et al., 1989). Over 160,000 people are estimated to be living with CF worldwide, with 1,000 new cases diagnosed each year (Guo et al., 2022). CFTR gene is located on chromosome 7, and mutations of this gene decrease the availability of the functional CFTR ion channel protein, which belongs to the ATP-binding cassette (ABC) family of proteins with transmembrane transport functions (Rommens et al., 1989). These genetic mutations elicit the synthesis of a dysfunctional CFTR protein associated primarily with a disruption in normal chloride (Cl^−^) and bicarbonate (HCO_3_^−^) ion transport (Anderson et al., 1991; Saint-Criq and Gray, 2017), which causes a lack of hydration in the extracellular mucus and secretions (Carrasco-Hernández et al., 2021). The functional impairment of mutated CFTR protein particularly affects the gastrointestinal and respiratory systems; however, other systems and organs are significantly involved (O’Sullivan and Freedman, 2009; Elborn, 2016). Currently, over 2000 CFTR mutations have been identified, and the most common (designated as F508del) is represented by a deletion of a phenylalanine at site 508 due to the genomic deletion of three nucleotides. CFTR mutations are currently grouped into six classes (I-VI) according to the cause of dysfunction (Marson et al., 2016): Class I—defective protein production, Class II—defects in CFTR processing, which includes the F508del mutation, Class III—impaired CFTR channel regulation, Class IV—reduced channel conductance, Class V—reduced number of channels present at the membrane, and Class VI—decreased of CFTR stability at the plasma membrane. The less severe forms of CF are those from Classes IV-VI, since a residual CFTR function (Elborn, 2016) is detectable in these groups. A functional defect in a CFTR protein and consequent reduced expression onto cell membrane alters chloride, thiocyanate, and bicarbonate transport across epithelial tissues, and negatively influences the biological activity in the intracellular compartments (Lukasiak and Zajac, 2021). It is noteworthy that a correlation between chloride anion transport and the acidification of intracellular vesicles has been reported (Barasch et al., 1988) along with defective acidification of the Golgi network, pre-lysosomes and endosomes due to impaired Cl-conductance in CF cells (Barasch et al., 1991). Lysosomes require correct acidification as the enzymes are activated at a low pH (Mindell, 2012), and in this case can be achieved with proper CFTR functioning, which contributes to a decrease in pH from an alkaline to an acidic state (Liu et al., 2012). CFTR dysfunction leads to alkalinization of intracellular vesicles resulting in ceramide accumulation and increased cell death, correlating with increase infection susceptibility (Teichgräber et al., 2008). Moreover, CFTR plays an important role in the maintenance of a low lysosomal pH in alveolar macrophages, which is the main condition to prevent bacteria growth (Swanson, 2006). CFTR modulates phagosome acidification in macrophages and is involved in bactericidal activity. Interestingly, Di et al. (2006) reported defective lysosomal acidification in CFTR−/− alveolar macrophages after fusion with phagosomes containing internalized bacteria, although this evidence was questioned (Haggie and Verkman, 2007). A further study addressed this issue (Deriy et al., 2009) demonstrating that a CFTR function deficiency negatively modulated the phagocytic response through defective acidification in the endosomal, lysosomal, and phagosomal compartments of alveolar macrophages. Defective myeloid cells’ function is a hallmark of CF, indeed the disease exhibits a new form of leukocyte adhesion deficiency, named LAD IV, as a consequence of a specific adhesion defect reported in monocytes (Sorio et al., 2016; Averna et al., 2021). More recent experimental analysis provide further support for a key role of the molecular alterations described in monocytes and, in general, of the innate immune system in the pathogenesis of CF, alterations that are known to play a role in the management of infective agents (Fan et al., 2022; Öz et al., 2022).

Defective CFTR elicits altered cellular redox state in epithelial cells and extracellular fluids, due to oxidative stress caused by an incorrect generation of reactive oxygen species (Lukasiak and Zajac, 2021). In addition, a loss of Ca^2+^ homeostasis is strongly associated with mitochondrial dysfunction and the disruption of endoplasmic reticulum (ER) function (Lukasiak and Zajac, 2021). Mitochondrial depolarization in CFTR-deficient cells elicits a dysfunction of the mitochondrial calcium uniporter (MCU) causing a reduced Ca^2+^ uptake (Antigny et al., 2009) and the disruption of mitochondrial structure. The MCU signals NLR family pyrin domain containing 3 (NLRP-3) activation via Ca^2+^, which supports the role of mitochondria in the development of inflammation in CFTR-defective cells (Rimessi et al., 2015; Rimessi et al., 2020). Misfolded CFTR proteins are retained in the ER where they cause ER condensation, IP3R (inositol triphosphate receptor) clustering, and Ca^2+^ release from ER stores. Moreover, the reduced expression or absence of CFTR at the plasma membrane level, leads to transient receptor potential cation channel (TRPC6) hyperactivity, which, in turn, increases the cytoplasmic Ca^2+^ level (Antigny et al., 2011).

CFTR impairment induces a major change in cellular proteostasis (Luciani et al., 2012; Villella et al., 2013). An increase in the generation of reactive oxygen species (ROS) can occur as a result of CFTR misfunction, leading to a post-translational modification of transglutaminase 2 (TG2), which is an enzyme able to catalyze several post-translational modifications of target proteins (Fesus and Piacentini, 2002; Nurminskaya and Belkin, 2012). In the case of a CFTR dysfunction, TG2 undergoes SUMOylation, which inhibits its ubiquitination and result in persistent activation and high TG2 protein levels (Luciani et al., 2009). This high activity of TG2 induces Beclin 1 (BECN1), a protein related to autophagy process that is involved in the formation of autophagosome (Kroemer et al., 2010), and several of its interactors to be sequestered into intracellular aggregates in the ER (Luciani et al., 2010; Villella et al., 2013; Villella et al., 2013). This BECN1 inactivation promotes the sequestration of a component of the BECN1 complex, phosphatidylinositol 3-kinase catalytic subunit type 3 (PIK3C3), which disrupts endosomal fusion/maturation and trafficking, and negatively impacts intracellular trafficking (Liang et al., 2008; Marat and Haucke, 2016). Therefore, aggresomal sequestration of BECN1 alters autophagy through defective autophagosome formation (Luciani et al., 2010; Villella et al., 2013).

### 1.2. Severe acute respiratory syndrome coronavirus-2

SARS-CoV-2 is a member of the *Betacoronavirus* genus, which also includes the human coronaviruses HCoV 229E (Bradburne et al., 1967), HCoV OC43 (Künkel and Herrler, 1993), HCoV HKU1 (Woo et al., 2005), HCoV NL63 (Van Der Hoek et al., 2006), SARS-CoV (Stadler et al., 2003), and Middle East respiratory syndrome (MERS-CoV) (de Groot et al., 2013). This virus is the etiological cause of the COVID-19 pandemic that was declared on March 11, 2020 (Wu et al., 2020). SARS-CoV-2-infected patients show symptoms of viral pneumonia, including fever, cough, and chest discomfort, and in severe cases, dyspnea and bilateral lung infiltration (Zhu et al., 2020). SARS-CoV-2 is a 30 kb single-stranded positive-sense RNA virus (Fuk-Woo Chan et al., 2020) with six functional open reading frames (ORFs) arranged in order from 5′ to 3′ as follows: replicase (ORF1a/ORF1b), spike (S), envelope (E), membrane (M), and nucleocapsid (N). In addition, seven putative ORFs encoding accessory proteins are interspersed among the structural genes (Hu et al., 2020; Piccaluga et al., 2022).

Viral entry is mediated by spike protein, that contains two non-covalently associated subunits: the S1 subunit mediates the attachment to the host cell membrane and engages angiotensin-converting enzyme 2 (ACE2) as the entrance receptor and the S2 subunit anchors the S protein to the membrane and mediates membrane fusion (Lebeau et al., 2020). Virus binding to ACE2 induces conformational changes in the S1 subunit exposing the S2′ cleavage site, which is cleaved by distinct proteases depending on the SARS-CoV-2 entrance route (Jackson et al., 2021). If the target cell expresses few TMPRSS2 or if virus–ACE2 complex cannot interact with TMPRSS2, the complex is internalized via clathrin-mediated endocytosis into the endolysosomes, where S2′ cleavage is performed by cathepsins, which require an acidic environment for their activity (Bayati et al., 2021). As the endosomal vesicle forms and clathrin detaches, the pH of the vesicle decreases and the endosome matures into a late endosome (pH of 5.5–6), which eventually fuses with the lysosome reaching a pH of 5–4.5 (Khan et al., 2020; Chen et al., 2022). In the presence of TMPRSS2, S2′ cleavage takes place at the cell surface and in both entry pathways, cleavage of the S2′ site reveals the fusion peptide and dissociation of S1 from S2 induces dramatic conformational changes in the S2 subunit, driving the fusion peptide forward into the target membrane, starting membrane fusion. Fusion between viral and cellular membranes forms a fusion pore with the subsequent release of viral RNA into the host cell cytoplasm (Jackson et al., 2021; Vkovski et al., 2021).

ORF1a and ORF1b are then translated into the pp1a and pp1ab polyproteins, which are cleaved into non-structural proteins (nsps) that form the viral replication and transcription complex (RTC) (Gupta et al., 2020). Along with nsps expression, viral replication organelles such as double-membrane vesicles (DMVs), convoluted membranes (CMs), and small open double-membrane spherules (DMSs), located in the perinuclear region of the host cells, create a favorable environment for viral genomic RNA replication and transcription of sub-genomic mRNAs (sg mRNAs). (Wolff et al., 2020; Vkovski et al., 2021). DMV formation appears to be caused by an altered autophagy response induced by the SARS-CoV-2 infection. The virus is able to elude and manipulate autophagy to fully promote its replication by increasing autophagosome formation, which is required for DMV formation, and for blocking autolysosome maturation (Qu et al., 2021; Liang et al., 2022).

Full-length positive-sense RNA is used as a template to produce both full-length negative-sense copies for genome replication and sgRNAs, the latter resulting from the discontinuous viral transcription process that produces a set of nested 3′ and 5′ co-terminal sgRNAs encoding for structural and accessory genes residing downstream of ORF1ab (Vkovski et al., 2021). Translated structural proteins than transit through the ER-Golgi intermediate compartment (ERGIC), where newly produced genomic RNA condensates and structural proteins interact, resulting in the assembly of viral particles (Scherer et al., 2022). Virions are secreted from the infected cell by exocytosis in two ways: by classical exocytosis through the Golgi compartment or by incorporation into deacidified lysosomes (Lebeau et al., 2020).

The first cells that SARS-CoV-2 targets during infection in humans are often the multiciliated cells in the nasopharynx or trachea or sustentacular cells in the nasal olfactory mucosa (Ahn et al., 2021). However, if the virus is not cleared by the induction of IFN-I or IFN-III, or B and T cell responses, it can spread to the lower respiratory tract by inhalation of virus particles from the upper respiratory tract or by gradual dissemination along the tracheobronchial tree (Hou et al., 2020; Huang et al., 2020; Salahudeen et al., 2020).

The infection of alveoli causes inflammation and restricts gas exchange (Katsura et al., 2020; Youk et al., 2020). SARS-CoV-2 infects alveolar type 2 (AT2) cells, which release the pulmonary surfactants that lubricate the lung and lower surface tension in the alveoli during respiration (Lumpuy-Castillo et al., 2020). In the adult human lung, AT2 cells also serve as progenitor cells of the alveolar type 1 (AT1) cells, which cover most of the alveolar surface and mediate gas exchange (Barkauskas et al., 2013; Katsura et al., 2020).

Since airways are the main target of SARS-CoV-2, the infection is generally transmitted through respiratory secretions and droplets generated by sneezing and/or coughing, although other transmission routes have been indicated, including through stool, sweat, and urine (Zhou et al., 2021).

The severity of COVID-19 is related to both the virulence of SARS-CoV-2 and the host immune response, with clinical manifestations ranging from asymptomatic to severe COVID-19 (Diamond and Kanneganti, 2022). SARS-CoV-2 infection can be asymptomatic, although most patients present with mild to moderate respiratory disease, and experience coughs, fevers, headaches, and myalgia (Huang et al., 2020; Zhang et al., 2020).

In severe cases, the most common symptom is dyspnea, which can progress to acute respiratory distress syndrome (ARDS), leading to respiratory failure (Carsana et al., 2020; Menter et al., 2020). ARDS is a form of lung injury characterized by inflammation and pulmonary vascular leakage (Tang et al., 2020). Patients with severe COVID-19 display hyperinflammation; secretion of pro-inflammatory cytokines due to a dysregulated immune response (Rebendenne et al., 2021): interleukin-1 (IL-1), IL-6, IL-8, and tumor necrosis factor (TNF), and elevated concentrations of inflammatory markers, including D-dimer, ferritin, and C-reactive protein (CRP) (Azkur et al., 2020; Del Valle et al., 2020; Goh et al., 2020; Zhang et al., 2020). IL-6 enlists immune mediators and induces cytokine release syndrome (CRS); in particular cytokines such as IFNγ, MCP1, IP-10, TNF-α, and IL-10 were found to be upregulated after SARS-CoV-2 infection, leading to local tissue damage and systemic non-protective inflammation inducing viral sepsis followed by lung injury and other complications like ARDS, pneumonitis, respiratory failure, sepsis shock, organ failure, and potentially, death (Giamarellos-Bourboulis et al., 2020; Li et al., 2020).

According to several studies, COVID-19 severity increases with age and the presence of certain comorbidities. At-risk populations are those with comorbidities such as hypertension, obesity, heart failure, cardiac arrhythmia, diabetes, kidney failure, and chronic pulmonary disease (Ng et al., 2021), and approximately 65% of people between the ages of 65 and 84 have one or more of these diseases and a loss in immune efficacy. Older patients display higher chances of respiratory failure and longer disease courses than younger patients after SARS-CoV-2 infections.

In addition, patients with higher severity have a greater likelihood of secondary bacterial, fungal, or viral infections than those with a less severe form of the disease. A poor COVID-19 outcome, including increased mortality, is related to the presence of either co-infection or superinfection. The most common bacteria detected in co-infections are *Klebsiella pneumoniae, Streptococcus pneumoniae*, and *Staphylococcus aureus*, while the most common viruses are influenza type A and type B, adenoviruses, and respiratory syncytial virus (RSV). Concerning superinfection, the most frequently found bacteria are *Acinetobacter* spp., *Escherichia coli*, and *Pseudomonas spp*, while the most common virus is rhinovirus (Fang et al., 2020; Mazzariol et al., 2021; Musuuza et al., 2021). An increase in multidrug-resistant bacterial infections has been observed in the context of COVID-19, which is a cause for concern that is probably due to the misuse of antibiotics (Sharma et al., 2022).

Children and adolescents seems to be less susceptible to SARS-CoV-2 infection (Lee et al., 2020) and in case of infection exhibit a broad range of clinical outcomes, with the majority having minimal to mild symptoms and rare hospitalization (Götzinger et al., 2020; Guan et al., 2020; Lu et al., 2020). Several hypotheses have been made to explain these observation: the lower ACE2 expression in children, which could lead to a lower susceptibility to SARS-CoV-2 infection, and the stronger innate and trained immune response compared to adults, which leads to a less pathogenic infection (Lu et al., 2020; Brodin, 2022). Some studies reported that the number and function of ACE2 receptors are lower in children than in adults, and lower ACE2 and TMPRSS2 expression in nasal and bronchial epithelial cells in children compared to adult COVID-19 patients was reported (Saheb Sharif-Askari et al., 2020); however, distinctions in the expression of viral entry factors between children and adults are not clear, as only some studies support the hypothesis that fewer ACE2 receptors in children can account for reduced viral entry into the lung (Chou et al., 2022). In addition, downregulation of ACE2 levels may expose the lungs to acute inflammation due to overexpression of angiotensin 2 (Bastolla et al., 2022). Some authors have also suggested that the measles/rubella/mumps vaccination could lead to fewer SARS-CoV-2 infections due to the homology between the fusion protein F domains of measles and mumps and a region of the S2 fusion protein of SARS-CoV-2 (Gao et al., 2015; Fidel and Noverr, 2020). Moreover, it has been hypothesized that pre-existing neutralizing antibodies and T cell immunity to commonly circulating human CoVs in younger age groups may cross-protect against SARS-CoV-2 (Verdoni et al., 2020; Piccaluga et al., 2021).

### 2. SARS-CoV-2 in cystic fibrosis patients: epidemiology

The majority of morbidity and mortality associated with CF is due to lung disease resulting from inflammation and chronic respiratory infections. Bacterial colonization and infection of the respiratory tracts of pwCF play an important role in the disruption of lung function. Several bacteria are involved in this process including *P. aeruginosa*, *H. influenzae*, *S. aureus*, *B. cepacia*, *S. maltophilia*, and *A. xylosoxidans* (Filkins and O’Toole, 2015). Viruses are also relevant in the pathogenesis of respiratory infections (i.e., influenza viruses, parainfluenza viruses, Rhinovirus, RSV) (Stelzer-Braid et al., 2012). Wat et al. (2008) showed that Influenza A and B and Rhinovirus are detected in approximately 28%–48% of CF patients with pulmonary exacerbations. RSV has also been shown to play an important role in pulmonary exacerbations, especially among young children (Somayaji et al., 2017).

Viral infections could have important effects on lung disease in pwCF, therefore, these patients were expected to be at increased risk of developing severe SARS-CoV-2 infections. However, several reports have demonstrated that worse outcomes did not occur in pwCF (Mathew et al., 2021; Biondo et al., 2022). A multinational cohort study of 40 individuals with CF identified an incidence of COVID-19 in pwCF of 0.07% compared to 0.15% in the general population (Cosgriff et al., 2020). Similarly, a multinational cohort study of 181 individuals with CF from 19 countries revealed that the outcome of SARS-CoV-2 infection for most pwCF could be less severe (McClenaghan et al., 2020). Nevertheless, hospitalization, oxygen therapy, intensive care, respiratory support, and death in lung-transplanted CF patients affected by COVID-19 were reported to be 2- to 6-fold more frequent than non-lung-transplanted pwCF (Jung et al., 2021). Indeed, a more severe clinical course may be associated with older age, CF-related diabetes, and lower lung function prior to infection, as well as with patients that have received an organ transplant.

Colombo et al. (2021) carried out a prospective multi center cohort study within the Italian Cystic Fibrosis Society involving 32 CF centers following 6,597 patients, reporting only few COVID-19 cases in pwCF, mainly adults, showing a relatively favorable COVID-19 course; moreover pwCF infected with SARS-CoV-2 evidenced no apparent effects on CF disease severity (Colombo et al., 2020, 2021). The most common COVID-19 symptoms in pwCF in that study were fever, cough, asthenia, and dyspnea, of which 50% were hospitalized and none was admitted to the intensive care unit (ICU). In a French study, that included 47 CF centers, 31 individuals with CF were diagnosed with COVID-19 and the risk of infection in this cohort was reduced by up to 93% with respect to the general population (cumulated incidence of 0.41%), with severe disease limited to a few post-lung transplant patients (Corvol et al., 2020). An observational retrospective study from Spain demonstrated that the incidence of COVID-19 in pwCF was 0.32% compared to 0.49% of the general Spanish population, with no deaths reported (Mondejar-Lopez et al., 2020).

Jaudszus et al. (2022) reported a cumulative incidence in a German population of SARS-CoV-2 of 8.3% in CF patients; however, infected patients did not experience more severe clinical courses and/or outcomes compared to those of the general population.

A retrospective study conducted on 532 CF patients from the Cystic Fibrosis Centre of Verona in the Veneto region of Italy reported a SARS-CoV-2 infection rate in the Veneto regional CF population of 0.19% at the beginning of the pandemic, compared to 0.40% in the general population. Only a single patient was positive for the SARS-CoV-2 infection, who reported only mild symptoms and did not require ICU admission (Bezzerri et al., 2020).

Published evidence indicated that pwCF do not experience higher risks of contracting SARS-CoV-2 infection compared to the general population, even though they are at risk of acute exacerbations often due to viral infections of the respiratory tract. Moreover, recent data suggest that COVID-19 disease outcomes in pwCF may be less severe than those caused by H1N1 infection during 2009 pandemic (Viviani et al., 2011; Bucher et al., 2016). Nevertheless, some observations also reported that some subsets within the CF population, including post-transplantation patients, show a more severe clinical outcome in case of SARS-CoV-2 infection.

### 3. SARS-CoV-2 in cystic fibrosis patients, an intricate interaction

Since retrospective epidemiological studies revealed that pwCF had lower SARS-CoV-2 infection incidences, milder symptoms, and lower risks of hospitalization, we first summarized the pwCF’s behaviors and treatments that were linked to favorable outcomes.

Several factors that may provide protection against SARS-CoV-2 in pwCF were considered. During the first stage of the pandemic, preventive measures were reinforced, such as face masks usage, careful hand hygiene, crowd avoidance, and patient segregation, whereas these processes had been previously established for pwCF (Cosgriff et al., 2020). In addition, the lower mean age of pwCF may be considered as a protective factor against a worse outcome in case of SARS-CoV-2 infection (Mondejar-Lopez et al., 2020).

Furthermore, drugs commonly prescribed to pwCF could attenuate COVID-19 severity; indeed, some of these drugs have been repurposed and are undergoing clinical trials.

Dornase alfa, a recombinant human DNase I enzyme, is commonly administered to pwCF as it facilitates airway clearance by disrupting mucus thickening caused by excessive formation of neutrophil extracellular traps (NETs). Patients with severe COVID-19 present an increase in inflammatory neutrophils and a mucus structure very similar to that of CF patients due to the accumulation of NETs. Since this accumulation can lead to ARDS, nebulized dornase alfa is currently in the clinical trials as a treatment for SARS-CoV-2-induced ARDS (Earhart et al., 2020; Okur et al., 2020). A study in which dornase alfa was administered to three people with COVID-19 demonstrated clinical improvements in oxygen saturation (SpO_2_), respiratory rate, and cough frequency and a decrease in NET formation and SARS-CoV-2 viral load after the treatment (Okur et al., 2020). Primary outcome from a randomized controlled trial revealed an improvement of ARDS from severe to moderate or from moderate to mild after dornase alfa treatment (Desilles et al., 2020).

In addition, corticosteroids are often utilized in the treatment of CF to reduce lung inflammation. These drugs may be useful in COVID-19 cases, as they downregulate pro-inflammatory genes such as NF-κB, thereby dampening immune and inflammatory responses. The results of several randomized trials support the protective effect of systemic corticosteroid therapy in hospitalized patients with COVID-19 that require supplemental oxygen (Sterne et al., 2020; Li et al., 2021). In contrast, corticosteroids have shown no benefits and may harm hospitalized patients with milder COVID-19 who do not require supplemental oxygen (Horby et al., 2021; Crothers et al., 2022). Therefore, WHO guidelines recommend the use of corticosteroids for hospitalized patients with severe COVID-19 disease who are in need of oxygen supplementation (Corticosteroids for COVID-19 [internet], 2020).

**Figure 1:**
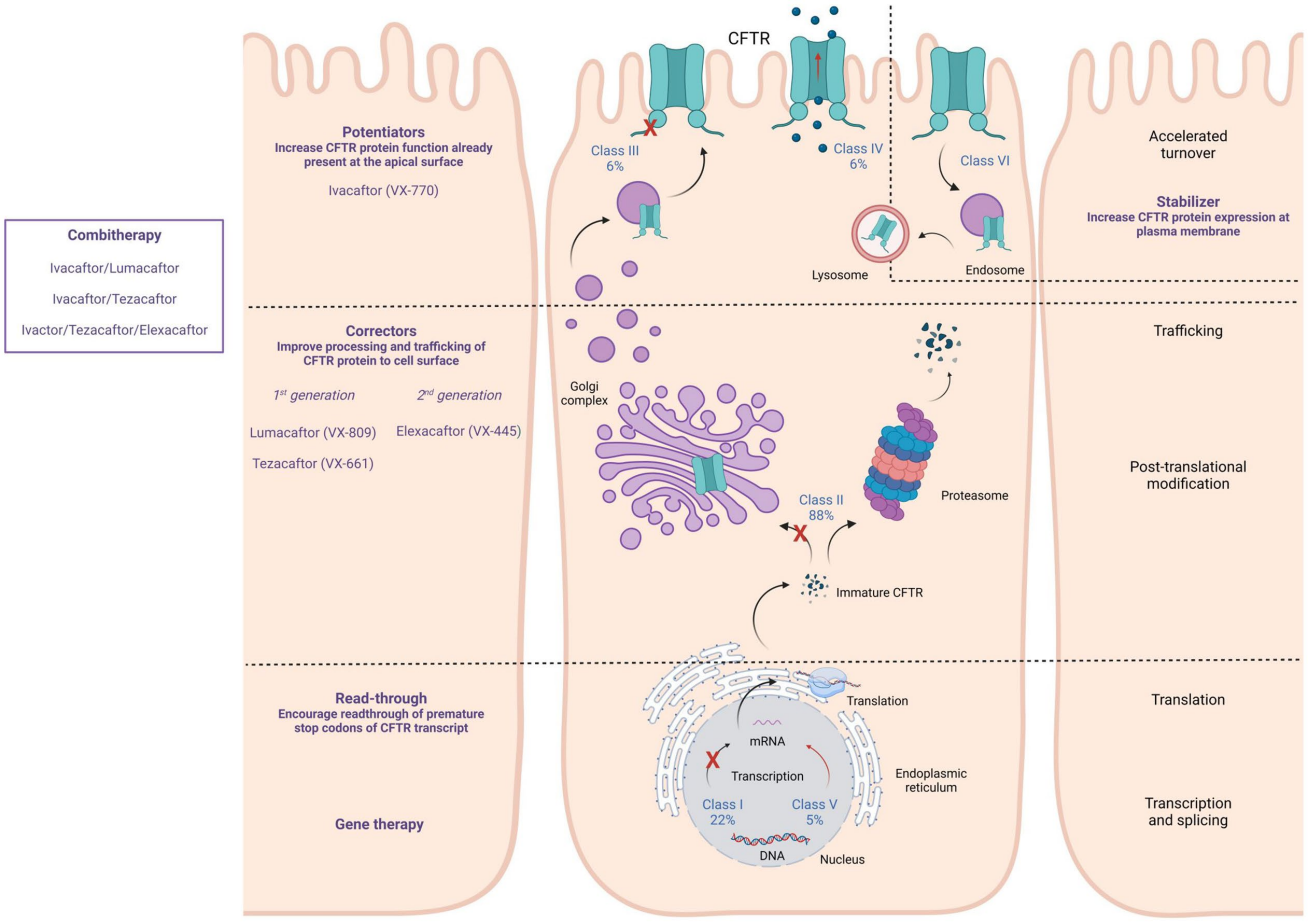
Available CFTR modulators, their specific action on different CFTR mutation classes, and their effects on CFTR expression, processing, and functioning. CFTR modulators can act by promoting the read-through of the premature stop codon of the CFTR transcript (Read-through), improving processing and trafficking of the CFTR protein (Correctors), increasing the CFTR protein function that is present at the apical surface (Potentiators), and increasing CFTR protein expression at the plasma membrane (Stabilizer).

CFTR modulators were developed as therapies that act upstream of the pathogenic cascade, and overcome the underlying dysfunctions caused by selected CFTR variants. These molecules are formulated to enhance or restore functioning and correct membrane localization of mutant CFTR proteins and are classified into five main groups depending on their effect on CFTR variants: potentiators, correctors, stabilizers, read-through agents, or amplifiers (Lopes-Pacheco, 2016; Figure 1). Currently, four compounds have been approved for the treatment of pwCF carrying specific CFTR mutations: the potentiator Ivacaftor (KALYDECO™ by Vertex Pharmaceuticals, Boston, Massachusetts, USA), which is the first CFTR modulator approved for pwCF; the corrector combinations Lumacaftor with Ivacaftor (ORKAMBI™ by Vertex Pharmaceuticals), Tezacaftor in combination with Ivacaftor (SYMDEKO™ or SYMKEVI ™ by Vertex Pharmaceuticals), and Elexacaftor with Ivacaftor and Tezacaftor (TRIKAFTA™ or KAFTRIO™ by Vertex Pharmaceuticals) (Ramsey et al., 2011; De Boeck et al., 2014; Wainwright et al., 2015; Rowe et al., 2017; Taylor-Cousar et al., 2017; Heijerman et al., 2019; Middleton et al., 2019). Results of a large cohort study revealed a protective effect in SARS-CoV-2 positive individuals under CFTR modulator therapy (ivacaftor and elexacaftor/tezacaftor/ivacaftor) since a significantly reduced need for oxygen was observed (Carr et al., 2022). In a population study by Simonson et al. (2022), pwCF that were on CFTR modulator therapy were observed in lower proportions in the COVID-19 positive group with symptomatic disease compared to patients who were not treated with this therapy (Simonson et al., 2022). A study by Bain et al. (2021) showed that children infected with SARS-CoV-2 who received CFTR modulator therapy were less likely to require hospitalization than those without CFTR modulator therapy.

### 4. SARS-CoV-2 infection and CFTR protein alteration

**Figure 2:**
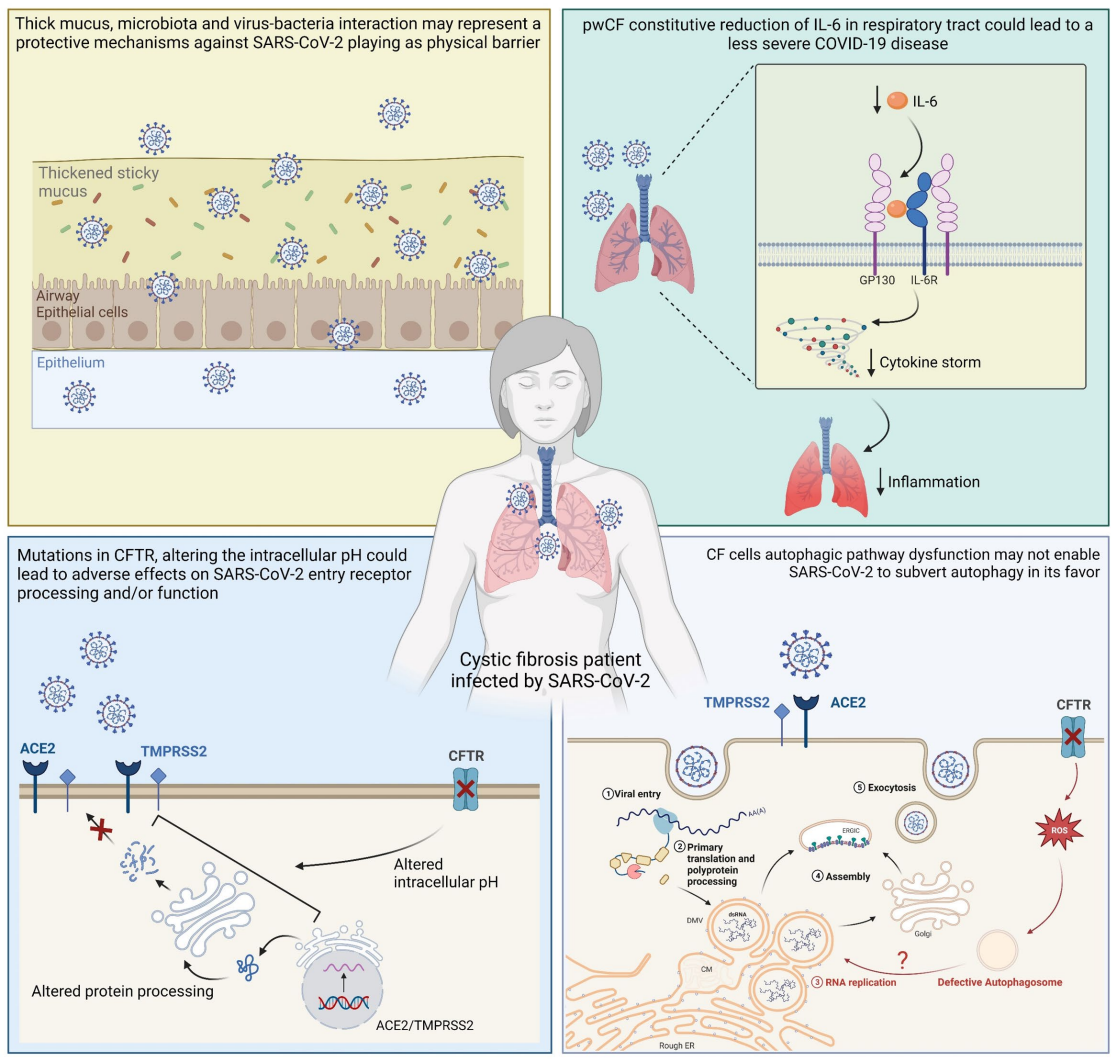
Graphical representation of suggested cellular mechanisms involved in reduced SARS-CoV-2 replication and consequent mild clinical course in pwCF. A physical barrier that serves as a defense against SARS-CoV-2 could be created by microbiota, thick mucus, and virus-bacteria interactions. pwCF constitutive reduction of IL-6 in the respiratory tract could lead to a less severe COVID-19 disease, and altered intracellular pH could lead to adverse effects on SARS-CoV-2 entry receptor processing and/or functioning. In addition, SARS-CoV-2 may not be able to manipulate autophagy to its advantage since the autophagic pathway in CF cells is dysfunctional.

Although the lifestyle and the commonly prescribed drugs of pwCF could play a role in protecting against severe SARS-CoV-2 infection, cellular and molecular mechanisms may be important factors in the lower infection rates and symptoms reported in pwCF (Table 1), suggesting that CFTR protein function and expression are actively involved in both ACE2-mediated virus uptake and SARS-CoV-2 viral particle replication once the virus has entered the host cells. Data on this subject are still scarce, although mechanisms suggested by the available literature could be found in the areas of immunology and virology or cellular and CF-specific molecular characteristics (Figure 2).

**Table 1:** Summary of possible mitigating factors of COVID-19 severity in pwCF.

Category	Possible mitigating factor	Effect on SARS-CoV-2 infection	Type of study	Citations
Preventive measures	Social distancing Use of face masks Careful hand hygiene	Limiting SARS-CoV-2 infection and spread	Epidemiological studies	Cosgriff et al. (2020)
CF therapies	Dornase alpha	Decrease in NETs formation and SARS-CoV-2 viral load	*In vivo* study	Earhart et al. (2020); Okur et al. (2020); Desilles et al. (2020)
	Corticosteroids	Downregulate pro-inflammatory genes mitigating the immune and inflammatory response	Randomized trials	Sterne et al. (2020); Li et al. (2021)
	CFTR modulators	Reduce COVID-19 severity	Epidemiological studies	Carr et al. (2022); Simonson et al. (2022); Bain et al. (2021)
Cellular and molecular mechanisms	Different ACE2/TMPRSS2 expression	Adverse effects on SARS-CoV-2 processing and/or function may reduce SARS-CoV-2 entry and replication	*In vitro* studies	Mondejar-Lopez et al. (2020); Stanton et al. (2020); Bezzerri et al. (2023); Bitossi et al. (2021); Sala et al. (2022); Ingraham et al. (2020)
	Thick respiratory secretions, respiratory microbiota, and virus-bacteria interactions	Mucus may be an important host barrier to the virus, as well as respiratory microbiota and virus-bacteria interaction may play a protective role	*In vitro* studies	Zanin et al. (2016); Wang et al. (2017); Shi and Gewirtz (2018); Mondejar-Lopez et al. (2020)
	Respiratory IL-6 reduction	Protective factor from severe SARS-CoV-2 infection-related cytokine storms	Retrospective studies	Marcinkiewicz et al. (2020)
	Defective autophagy	Dysfunction in autophagic pathway of CF cells not enabling the virus to subvert autophagy in its favor	*In vitro* studies	Gies et al. (2020); Gassen et al. (2021); Merigo et al. (2022)
	Deregulation in CFTR expression/function	Ionic and pH alteration of organelles may alter SARS-CoV-2 replication	*In vitro* studies	Lotti et al. (2022)
	Cell architecture alteration	Cellular organelles structural alteration may affect SARS-CoV-2 replication capability	*In vitro* studies	Merigo et al. (2022)

Studies have suggested that thick respiratory secretions could indicate a more difficult viral infection, such as that from the influenza virus where mucus appears to be an important host barrier to the virus (Zanin et al., 2016; Wang et al., 2017). In addition, other studies have indicated that respiratory microbiota and virus-bacteria interactions in the lungs of pwCF may be protective mechanisms against respiratory virus infection (Shi and Gewirtz, 2018; Mondejar-Lopez et al., 2020). Furthermore, the relationships among microbiota, virus, and bacteria superinfections could require further investigation, since Cosgriff and co-workers suggested that pwCF are generally meticulous in controlling their exposure to microbes, and although this does not fully explain the better outcome than expected after SARS-CoV-2 infection, it could justify the low number of SARS-CoV-2 infections (Cosgriff et al., 2020).

Elevated IL-6 levels are associated with severe disease and mortality in COVID-19 patients; however, pwCF show a constitutive reduction of IL-6 in the respiratory tract, which could serve as a protective factor from severe SARS-CoV-2 infection-related cytokine storms (Marcinkiewicz et al., 2020). In terms of cellular mechanisms, CF cells may be less prone to SARS-CoV-2 infection (Lotti et al., 2022). ACE2 acts as a cellular receptor for SARS-CoV-2 and several studies have focused on the role of mutated CFTR in the regulation of ACE2, although results are conflicting. Mutations in the CFTR gene may alter the protein abundances of ACE2 and TMPRSS2, limiting susceptibility to SARS-CoV-2 infection and mitigating the consequent damage to the lungs (Mondejar-Lopez et al., 2020). In particular, the impairment of CFTR function and expression alters the pH of organelles involved in the protein secretory pathway, which could negatively affect ACE2 and glycosylation, processing and/or functioning, thereby reducing SARS-CoV-2 entry and replication (Stanton et al., 2020; Bezzerri et al., 2023). A recent study evidenced that ACE2 mRNA and protein expression was significantly reduced in both primary human bronchial and nasal epithelial cells isolated from CF patients compared to healthy tissue. Moreover, it was observed that mutated CFTR channel could induce mislocalization of ACE2 into the ER and that, depending on the pattern of ACE2 expression, SARS-CoV-2 S protein induced high levels of IL-6 in healthy primary cells, but a very weak response in primary CF cells (Bezzerri et al., 2023). Bitossi et al. (2021) showed reduced levels of ACE2, furin, and TMPRSS2 in the oropharyngeal cells of CF patients compared with those of healthy individuals (Bitossi et al., 2021). In contrast, an RNA-seq analysis by Sala et al. (2022) revealed ACE2 expression was similar in the nasal epithelia of healthy people and pwCF. Another recent study based on gene microarray data compared the expression of ACE2 and TMPRSS2 mRNA levels in the airway epithelial cells of CF and non-CF patients and observed higher ACE2 mRNA levels and lower TMPRSS2 levels in the CF cells than those in the non-CF cells (Stanton et al., 2020). A higher concentration of ACE2 could lead to increased binding of SARS-CoV-2 to host cells, as well as a higher rate of angiotensin 2 (ANG-2) cleavage into the anti-inflammatory angiotensin 1–7 [ANG-(1–7)] (Ingraham et al., 2020), which could contribute to a lower severity of SARS-CoV-2 infections in pwCF.

**Figure 3:**
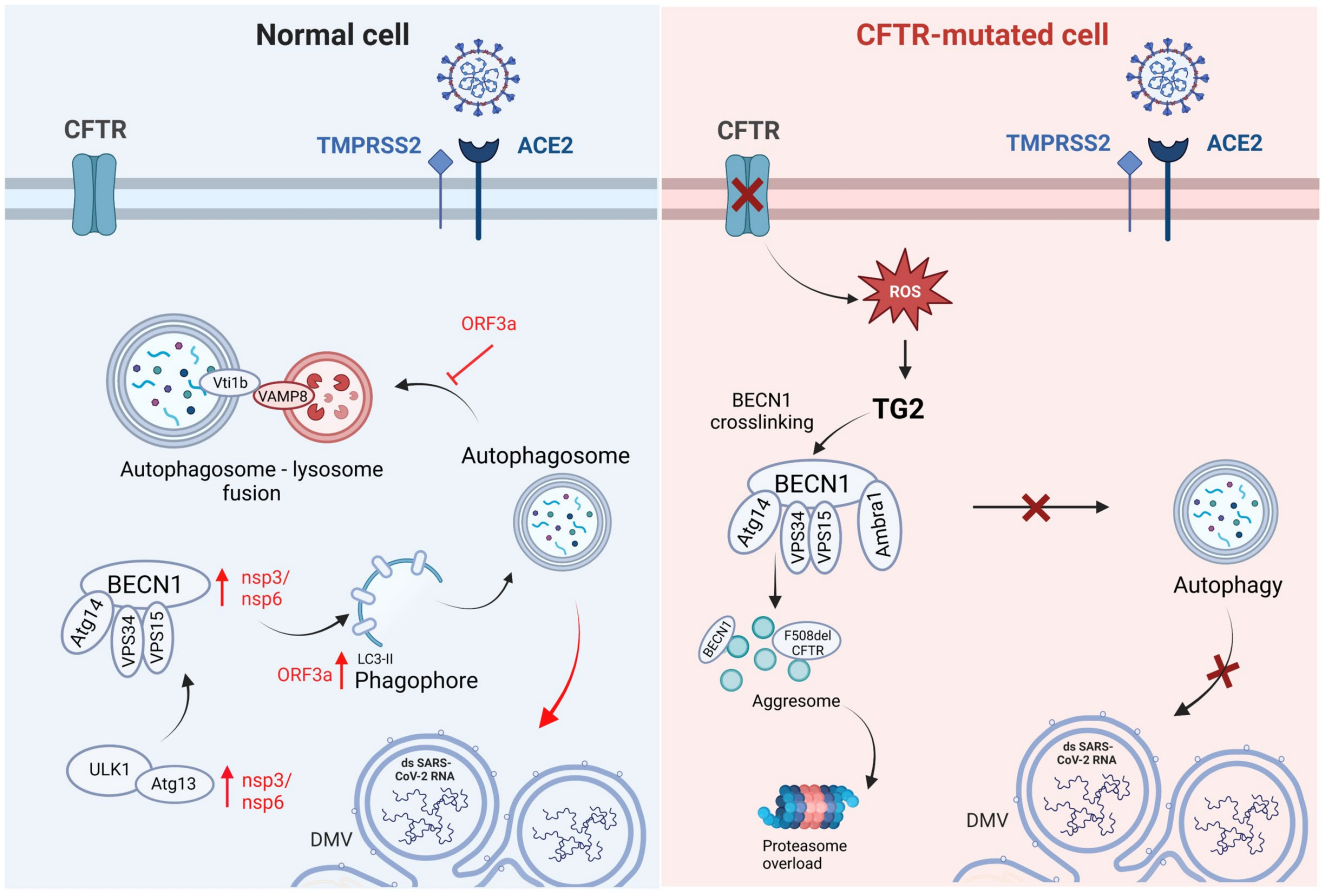
Graphical representation of autophagic pathway differences in Normal vs. CFTR-mutated cells and the respective involvement of SARS-CoV-2 infection. In normal cells, SARS-CoV-2 induces autophagosome formation to favor its replication using nsp3/6 and ORF3a, with the latter blocking autophagosome-lysosome fusion. In CF cells, the reduced SARS-CoV-2 replication could be related to the defective autophagic pathway. CFTR: cystic fibrosis transmembrane conductance regulator; TMPRSS2: transmembrane serine protease 2; ACE2: angiotensin converting enzyme 2; Vti1b: vesicle transport through interaction with T-SNAREs 1B; VAMP8: vesicle associated membrane protein 8; ORF3a: open reading frame 3a; Atg14: autophagy related 14; VPS15: vacuolar protein sorting 15; VPS34: vacuolar protein sorting 34; nsp3,6: non-structural protein 3,6; ULK1: Unc-51-like kinase 1; Atg13: autophagy related 13; DMV: double-membrane vesicle.

Considering these discordant studies on the role of ACE2, mechanisms other than viral entrance could be involved in the lower SARS-CoV-2 replication and milder COVID-19 symptoms reported in pwCF. Recent *in vitro* studies demonstrated significantly lower SARS-CoV-2 infection rate in CFTR-defective cells than in normal cells, demonstrating that alteration or complete deletion of the CFTR gene induced a significant decrease in SARS-CoV-2 content (Lotti et al., 2022; Lagni et al., 2023). These studies supported the involvement of CFTR function and expression in the replication of SARS-CoV-2, and demonstrated the efficacy of CFTR-inhibitors (IOWH-032 and PPQ-102) in the reduction of viral load in WT cells, significantly more effective in the viral post-entry phase, strengthening the suggestion of a specific involvement of the CFTR protein function in the viral life cycle. It has also been demonstrated *in vitro* that the recovery of CFTR expression and function by CFTR modulators treatment is associated with a sudden increase in viral replication (Lotti et al., 2022), in contrast to clinical observation reporting a less severe outcome of COVID-19 in pwCF in case of CFTR modulators therapy (Bain et al., 2021; Simonson et al., 2022). Bringing together the conflicting results of *in vitro* and clinical studies, it could be suggested that the only partial recovery of CFTR function recorded in patients lead to an improvement of clinical symptoms enabling the patient to tackle potential infections more effectively, but it does not directly affect *in vitro* SARS-CoV-2 replication capability that might depend also on other biochemical mechanisms associated to the CF phenotype. As previously reported in this review, CF leads to BECN1 inactivation, which inhibits endosomal fusion/maturation and trafficking and negatively impacts intracellular trafficking (Liang et al., 2008; Marat and Haucke, 2016), thereby altering autophagy through defective autophagosome formation (Luciani et al., 2010; Villella et al., 2013). Autophagy can be detrimental for viral infections, since it is involved in antiviral innate immunity, selectively targeting pathogens for degradation, promoting pathogen recognition, antigen presentation and inflammatory cytokine responses, and controlling cell survival (Levine et al., 2011; Ahmad et al., 2018; Choi et al., 2018). However, several viruses have been reported to subvert this cellular machinery to favor their replication and release (Choi et al., 2018). Coronaviruses require the formation of double-membrane vesicles to aid replication and transcription of the virus; therefore, it was suggested that SARS-CoV-2 may manipulate the autophagosomal machinery to facilitate the formation of double-membrane vesicles (Randhawa et al., 2020), subverting autophagy in its favor to accelerate its replication (Gassen et al., 2019; Fakher et al., 2020; Sargazi et al., 2021). Several SARS-CoV-2 genes and proteins appear to be involved in autophagy regulation (Figure 3): NSP3 and NSP6 proteins inhibit the Akt–mTOR pathway activate the ULK-1-Atg13 and VPS34-VPS15-Beclin1 complexes, and induce the formation of autophagy-specific phagophores, which result in the activation of the autophagy pathway (Cottam et al., 2011; Mihelc et al., 2020; Fattahi et al., 2022); ORF3a has the dual effect of enhancing the phosphatidylethanolamine (PE)-dependent conversion of non-lipidated LC3-I to active LC3-ll that is incorporated in the extending phagophore membrane, leading to its elongation into complete autophagosomes, and blocking autophagosome maturation and fusion with lysosomes by inhibiting the assembly of the VAMP8 (a significant vesicle adaptor protein) complex through the regulation of a membrane fusion accelerator (such as HOPS, VPS39, or STX17) (Miao et al., 2021; Qu et al., 2021; Zhang et al., 2021). Therefore, SARS-CoV-2 appears to induce autophagosome formation to favor its replication and block autophagosome-lysosome fusion (Qu et al., 2021; Zhang et al., 2021). Shang et al. (2021) revealed that by inhibiting autophagy with 3-MA, an inhibitor of class I PI3P kinase and VPS34, viral replication was significantly reduced. Gies et al. (2020) concluded that chloroquine inhibits *in vitro* SARS-CoV-2 replication by impairing autophagosome-lysosome fusion, while Gassen et al. (2021) demonstrated that AMPK activators, which lead to autophagy assembly and initiation, were associated with increased viral replication.

The correct formation of the autophagosome is necessary for SARS-CoV-2 replication since its inhibition results in reduced viral replication. In the context of CF, reduced SARS-CoV-2 replication could depend on defective autophagosome formation (Figure 3). A recent study by Merigo et al. (2022) focused on the ultrastructural analysis of bronchial epithelial cells after SARS-CoV-2 infection and reported that autophagosomes containing replicative structures were observed at a late time of infection (from 48 h post-infection). Isogenic bronchial epithelial cells expressing deficient and malfunctioning CFTRs displayed autophagosomal structures during both the early and late time of infection containing cellular recycling material, as opposed to replicative structures. Moreover, it is well known that viruses such as SARS-CoV-2 are able to induce DMVs to replicate their genome sheltered from innate immunity mechanisms. In this context, the ER and the autophagic processes, as well as the Golgi apparatus, mitochondria and lipid droplets, have been held essential for the formation of DMVs induced by SARS-CoV-2. Since these processes and cellular components are altered due to impaired anions conductance in CF cells, the connection between CFTR dysfunction and DMVs alteration is highly likely. Indeed, it has been reported that CFTR-modified cells do show some differences in cellular structure compared to normal cells and once infected with SARS-CoV-2 they display an impaired organization of replicative structures (Merigo et al., 2022).

It is important to note that all of these *in vitro* studies have been performed on models carrying F508del mutation, the most common mutation in pwCF. However, it is possible to hypothesize that different classes of mutation and thus a different severity of CFTR dysfunction may be related to a different impact on SARS-CoV-2 replication.

## 5. Conclusion

In addition to bacterial colonization and infection of the CF respiratory tract by *P. aeruginosa*, *H. influenzae*, *S. aureus*, *B. cepacia*, *S. maltophilia*, or *A. xylosoxidans* (Sibley et al., 2008; Filkins and O’Toole, 2015; Billard et al., 2017), several viruses (i.e., influenza viruses, parainfluenza viruses, and RSV) can play important roles in the pathogenesis of respiratory infections (Stelzer-Braid et al., 2012). Although pwCF and non-CF people are equally affected by viral respiratory infections, the severity is increased in pwCF (Stelzer-Braid et al., 2012); it is noteworthy that hospitalization of pwCF is concomitant or posterior to viral infection.

Viral pathogens were found in 13% to 52% of CF patients, with a higher percentage in younger people than older patients. Viral infection was found in 40% of cases that showed pulmonary exacerbation and required hospitalization, compared to 9% of cases with stable clinical statuses (Frickmann et al., 2012). Respiratory syncytial viruses (RSV) and rhinoviruses comprised 9 to 58% of all reported viruses with the latter being the most common in young children and those requiring continuous oxygen therapy (Burns et al., 2012; Billard et al., 2017). Rhinoviruses were found in 58% of individuals with respiratory symptoms, and a high viral load was linked to severe upper and lower respiratory tract infections (Wark et al., 2012; Billard et al., 2017). Influenza A and B can affect 12–27% of people, showing up to 77%. These viruses are associated with acute respiratory illnesses that dramatically raise the risk of hospitalization and cause severe manifestation in CF patients (Hordvik et al., 1989; Wark et al., 2012).

Therefore, although respiratory tract viral infections frequently cause acute intensifications of chronic lung illnesses in pwCF, published research suggests that incidence rates of SARS-CoV-2 infection are lower in pwCF than those in the general population, better clinical outcomes are observed in pwCF than those expected, and a lower rate of hospitalization is evident. Evidence also suggests that although specific CF population subsets, such as those post-transplantation, may experience a more severe clinical course, this does not represent the majority of CF patients who are exposed to specific pharmacological treatments necessary to control transplant rejection. The reported low incidence of SARS-CoV-2 infection among pwCF is likely a result of several variables, including the effective application of infection control practices that are part of regular CF care as well as the use of drugs that have antiviral effects. Nevertheless, infections that occur are relatively benign, suggesting that other factors are involved. These factors could include cellular and molecular alterations linked to CFTR dysfunction and the consequent expression of virus receptors, as well as ionic and pH alterations. Any of these could represent the major factor inducing this unusual response to SARS-CoV-2 infection in pwCF, but they also might need to act together. A detailed analysis of the molecular mechanisms is essential to clarify the pathways involved in this relationship between SARS-CoV-2 infection and pwCF to understand the viral life cycle and suggest potential new virus interference strategies.

## Author contributions

DG conceived the study. VL and AL elaborated the figures and tables, made the literature review, and wrote the manuscript. VL, AL, ED, CS, and DG assisted the writing and revision of the manuscript. All authors contributed to the article and approved the submitted version.

## Funding

The present study was supported by Fondazione Cariverona, ENACT project VIRO-COVID and by Lega Italiana Fibrosi Cistica onlus.

## Conflict of interest

The authors declare that the research was conducted in the absence of any commercial or financial relationships that could be construed as a potential conflict of interest.

## Publisher’s note

All claims expressed in this article are solely those of the authors and do not necessarily represent those of their affiliated organizations, or those of the publisher, the editors and the reviewers. Any product that may be evaluated in this article, or claim that may be made by its manufacturer, is not guaranteed or endorsed by the publisher.
